# Happiness in action: the impact of positive affect on the time of the conscious intention to act

**DOI:** 10.3389/fpsyg.2015.01307

**Published:** 2015-09-01

**Authors:** Davide Rigoni, Jelle Demanet, Giuseppe Sartori

**Affiliations:** ^1^Department of Experimental Psychology, Ghent UniversityGhent, Belgium; ^2^Department of General Psychology, University of PaduaPadua, Italy

**Keywords:** intention, positive affect, action control, conscious awareness, intentional action, Libet task

## Abstract

The temporal relationship between our conscious intentions to act and the action itself has been widely investigated. Previous research consistently shows that the motor intention enters awareness a few 100 ms before movement onset. As research in other domains has shown that most behavior is affected by the emotional state people are in, it is remarkable that the role of emotional states on intention awareness has never been investigated. Here we tested the hypothesis that positive and negative affects have opposite effects on the temporal relationship between the conscious intention to act and the action itself. A mood induction procedure that combined guided imagery and music listening was employed to induce positive, negative, or neutral affective states. After each mood induction session, participants were asked to execute voluntary self-paced movements and to report when they formed the intention to act. Exposure to pleasant material, as compared to exposure to unpleasant material, enhanced positive affect and dampened negative affect. Importantly, in the positive affect condition participants reported their intention to act earlier in time with respect to action onset, as compared to when they were in the negative or in the neutral affect conditions. Conversely the reported time of the intention to act when participants experienced negative affect did not differ significantly from the neutral condition. These findings suggest that the temporal relationship between the conscious intention to act and the action itself is malleable to changes in affective states and may indicate that positive affect enhances intentional awareness.

## Introduction

A key feature of voluntary movements is the conscious experience of the intention to act, which can be described as the feeling that we are planning to do something (Haggard, 2005). Previous research in experimental psychology and cognitive neuroscience mainly focused on the so-called motor intention, on its temporal relationship to movement execution and to related brain potentials (for a review see Haggard, 2008). In the pioneering study of Libet et al. (1983), participants had to perform simple self-paced manual movements while watching a rotating clock hand that was displayed on a screen. After executing the movement, they were asked to report, by using the clock hand, when they felt the first urge or intention to execute the movement, the so-called W-moment. It was found that intentions were reported by the subjects ∼200 ms before the onset of the actual movement, and up to ∼2 s after the onset of movement-related brain potentials, such as the readiness potential (Shibasaki and Hallett, 2006). Although both the validity of the W-moment as a reliable measure of the timing of the intention and the interpretation of the readiness potential as reflecting motor preparation have been criticized (Gomes, 1998; Trevena and Miller, 2010; Schurger et al., 2012), following studies have replicated the main finding (Haggard and Eimer, 1999; Soon et al., 2008; Rigoni et al., 2013). For instance, Haggard and Eimer (1999) provided evidence that the W-moment is linked to the lateralized readiness potential, which reflects activity over motor areas – including primary motor cortex – contralateral to the responding hand. More recently, it was found that the W-moment is often reported after muscular activation of the responding hand (Rigoni et al., 2013). These observations have been taken as evidence that awareness of a motor intention, as measured during the Libet task, is a latecomer during movement preparation and execution. The conscious intention to act would reflect the moment at which people become aware that they are about to execute a voluntary movement, rather than the ‘driving force’ of our actions (Hallett, 2007). Awareness of the motor intention presumably arises when neural activity in specific brain circuits – including the premotor cortex, the supplementary motor areas, and the posterior parietal cortex (Lau et al., 2004; Desmurget and Sirigu, 2009) – exceeds an individual’s threshold level, and therefore only after specific movement-related brain processes occurred unconsciously.

While previous studies shed lights on neural (Libet et al., 1983; Lau et al., 2004, 2007; Rigoni et al., 2013) and psychological (Banks and Isham, 2009; Rigoni et al., 2010) determinants of intention awareness, little is known about the impact of different affective states on intention awareness. Yet human actions are almost always accompanied by specific moods or emotions: a child is likely to feel happy and excited while grasping his or her favorite chocolate bar; conversely, we probably feel angry and frustrated while grasping the phone to inform our colleagues that we will be late for the scheduled meeting because of a traffic jam. In some extreme circumstances, very intense emotional experiences can also have an impact on the experience of conscious control one has over one’s own behavior. For instance, in the so-called ‘crime of passion,’ the perpetrator commits a violent act, like a murder, against someone because of a sudden and strong rage or heartbreak. The use of this type of crime as a defendant’s excuse for committing the crime was recently restored in the UK (Whitehead and Hough, 2012) based on the argument that, in such circumstances, actions can be considered to be un-intentional because they are characterized by the experience of a temporary loss of conscious control.

The link between intentional control and affective state was recently investigated in a study of Aarts et al. (2012). In this study, evidence was found that sense of agency, which is the subjective experience of controlling one’s actions and their subsequent effects, is stronger when positive emotional pictures were presented. Conversely, the sense of agency is reduced for actions resulting in negative emotional outcomes (Yoshie and Haggard, 2013). This suggests that subjective experiences surrounding intentional control can be modulated by affective stimuli. However, besides anecdotic observations, to our knowledge, no studies have directly investigated the impact of positive or negative affective states on intention awareness.

A large body of literature already indicated that positive and negative affective states have opposite effects on different levels of cognitive processing. In a nutshell, different theories have suggested that positive affect has a broadening function for cognition, whereas negative affect is supposed to narrow cognitive processes (Easterbrook, 1959; Derryberry and Tucker, 1994; Fredrickson, 2001, 2004). Positive affect has been shown to enhance flexibility in problem solving (Isen et al., 1987) and to expand the scope of semantic memory in word association tasks (Isen and Daubman, 1984; Isen et al., 1985; Ashby et al., 1999). These broadening effects of positive affect have been suggested to originate from changes in for more basic cognitive functions, such as selective attention (Rowe et al., 2007). For instance, Rowe et al. (2007) induced affective changes in their participants by means of pleasant and unpleasant music. Then participants performed the Flanker task, an experimental paradigm requiring participants to focus on a central target and ignore the irrelevant flankers presented laterally. They found that positive affect influenced the ability to selectively focus on the target by increasing the processing of the spatially distant flanking distracters. Although this observation suggests a detrimental effect of positive affect for tasks requiring a narrow and focused selective visual attention, they are consistent with the hypothesis that positive affect increases the breadth of the attentional focus (Fenske and Eastwood, 2003; Rowe et al., 2007). However, while affect-related changes in attention have been reported elsewhere (e.g., Chajut and Algom, 2003; Grol et al., 2014) this effect was not always replicated with other conflict-tasks (Martin and Kerns, 2011; van Steenbergen et al., 2011; Bruyneel et al., 2013). Further research has shown that positive affect can also reduce ‘attentional blink’ – i.e., the inability to detect a stimulus presented in a sequence when this is presented shortly after the previous stimulus – by increasing attention resources (Olivers and Nieuwenhuis, 2014). In addition, Soto et al. (2009) found that positive affect induced by listening to pleasant music, enhanced visual awareness in patients with visual neglect, suggesting that positive affect can decrease visual neglect by increasing attentional resources to the external space.

The studies described above suggest that positive affect, as compared to negative affect, broadens awareness by enhancing the allocation of attentional resources to both internal (e.g., semantic memory) and external stimuli. Whether positive affect can also impact on the awareness of inner states during the execution of intentional actions has never been investigated directly. Partial support for the hypothesis that intention awareness can be modulated by the valence of individuals’ affective state comes from a recent study on expert meditators (Jo et al., 2015). Meditation is a psychological state that is known to decrease negative affect, such as anxiety, and simultaneously increase positive affect (Davidson et al., 2003). Jo et al. (2015) employed the Libet’s task to investigate whether meditation leads to changes in intention awareness, and found that motor intentions were reported earlier in time in meditators as compared to non-meditators. This observation was taken as evidence that meditators are more capable to access inner processes underlying the initiation of a voluntary movement (Jo et al., 2015). It should be noted, however, that affect or mood were not directly manipulated in Jo et al. (2015) study, and thus the question whether affective states alone impact on intention awareness remains unanswered.

In the current study, we wanted to investigate whether different affects can influence awareness of inner motor states, such as when we attend to our own motor intentions. If positive affect broadens the scope of attention by enhancing attentional resources, the motor intention should be detected earlier in time when individuals experience positive affect as compared to when they experience negative affect. To test this hypothesis, a previously validated mood induction procedure (Mayer et al., 1995) was employed to trigger temporarily positive, neutral, or negative affective states in a group of healthy participants. After each mood induction session, participants performed the Libet task (Libet et al., 1983), where they were asked to attend to their own intentions and report when they formed the conscious intention to perform the movement. Our specific hypothesis was that, as compared to negative affect, positive affect should broaden intention awareness, and this should be reflected in earlier access to internal states related to movement preparation (Ganos et al., 2014).

## Materials and Methods

### Experimental Design

The study included a full within-subjects experimental design with the type of induced mood (Positive, Negative, and Neutral) as within-subjects variable. The effect of each mood induction was checked by means of a Dutch version of a 16-items adjective scale (Mayer et al., 1995; see below for more details). The reported time of conscious intention (i.e., W-moment) during the Libet task was entered as dependent variable in the analyses.

### Participants

Thirty-two undergraduate students of Ghent University (20 females, 12 males), aged 18–30 years (*M* = 19.81) participated to the experiment in exchange of course credits. Six left-handed participants took part to the experiment and carried out the main task with their dominant hand (see Experiment Description below). Participants had no previous neurological or psychiatric history, and they had normal or corrected-to-normal vision. Informed consents were obtained prior the experiment. The study was conducted according to the Declaration of Helsinki and was approved by the local ethic committee.

### Experimental Procedure

At the beginning of the experiment, participants received detailed instructions about the experimental procedure as well as about the Libet task (Libet et al., 1983). After practicing the Libet task (i.e., five trials), a baseline measurement of each individual’s affective state was assessed by means of a Dutch version of a 16-items adjective scale (Mayer et al., 1995) for happiness (cheerful, happy, lively, joyful), anger (angry, furious, mad, hostile), fear (scared, fearful, afraid, nervous), and sadness (blue, depressed, unhappy, sad). Each of the 16 adjectives was responded to on a 4-point Likert scale ranging from 1 = *definitely do not feel* to 4 = *definitely feel*. The experiment included three mood induction sessions, each followed by a self-report measurement of participants’ affective states (Mayer et al., 1995) and the Libet task. After performing the Libet task, participants were given 2 min to try to get their affective state back to normal. Each mood induction session lasted 4 min and combined a guided imagery and a music procedure to induce positive, negative, or neutral affective state. The order of the mood induction session was randomized across participants. Stimuli presentation and response collection were controlled by E-Prime 2.0 software (Psychology Software Tools, 2013). The experimental session lasted about 1 h.

### Mood Induction

We combined a guided imagery and a music procedure to induce positive, negative, and neutral affective states in each participant (Mayer et al., 1995; Mitchell and Phillips, 2007; Robinson et al., 2012). For each mood induction session, participants were shown a series of eight scenarios that remained on the screen for 30 s. They were instructed to get into the mood suggested by the scenarios and relate them to situations in their own life. They were also encouraged to display outwardly the emotions evoked and to get as deeply into the emotion as possible. While reading the sentences, participants wore around-ear headphones and listened to a music track that was previously selected to induce positive, negative, or neutral affective state. To induce positive and negative affective states, we employed the happiness- and anger-inducing scenarios used in Mayer et al. (1995) study and translated into Dutch. Positive scenarios were presented as light red text on a yellow background and were meant to induce pleasant emotions such as happiness (e.g. “*It’s your birthday and friends throw you a terrific surprise party*,” “*You wake up on a Saturday after a number of wintry-cold rainy days, and the temperature is in the high sixties.*”). Conversely, negative scenarios were presented as light gray text on a dark blue background and were meant to induce unpleasant emotions, such as anger (e.g., “*Somebody files a false legal claim against you*,” “*A student stole the exam in an important course you’re taking. The professor takes it out on everyone by making such a tough exam that you get a very low grade even though you understood the material.”*). Neutral scenarios (Velten, 1968) featured black text on a white background and were assumed to induce neither positive nor negative affective states (e.g., “*The Pacific Ocean has fish*,” “*The Appalachian Highlands are worn down mountains and plateaus stretching from the northern Alabama to the St. Lawrence River in Canada.*”). While reading the positive scenarios participants listened to ‘*A little night music*’ by Wolfgang Amadeus Mozart. Conversely, while reading the negative scenarios they listened to ‘*Night on bald Mountain*’ by Modest Mussorgski. These two music tracks have been used previously to induce positive and negative affective states, respectively (Vastfjall, 2002; Robinson et al., 2012). While reading the neutral scenarios participants listened to ‘*Neptune*’ by Gustav Holst (Vastfjall, 2002; Robinson et al., 2012).

### Libet Task

Participants performed 30 trials of the Libet task (Libet et al., 1983) after each mood induction session, for a total of 90 trials. At the beginning of each trial a cursor appeared at a random position around a clock face and started to rotate clockwise around the clock, completing one full rotation in 2540 ms. The clock was 40 mm in diameter and was composed by 60 evenly spaced yellow spots on a black background. For all the duration of the task participants were asked to fixate the center of the clock – i.e., a light blue cross – and to rest the index finger of their dominant hand on the response button, which was the keyboard spacebar. They were encouraged to press the button spontaneously and suddenly at a time of their own choosing, following at least half rotation of the cursor. They were asked not to pre-plan the time of the button press and were told that they could choose not to make a button press in any trial. After the button press the cursor continued rotating for a random interval between 800 and 1500 ms and then stopped. At that time participants were to act on the mouse with their non-dominant hand and click at the “time” (i.e., exact position on the clock) when they formed the intention or decision to respond – e.g., the time at which they decided “*to press the button now*” (i.e., W-moment).

### Data Analysis and Statistics

Trials of the Libet task where participants provided no response or where they did not wait for half rotation of the clock – i.e., 1270 ms – before pressing the button were discarded from data analysis (12.4% of total trials). Three participants were excluded from further analyses because of the high rejection rate – i.e., more than 66% rejected trials in at least one experimental condition. Analyses were therefore conducted on the remaining 29 participants. The effect of the mood induction on intention awareness was tested by means of a repeated measure ANOVA with the type of mood induction (Positive, Neutral, Negative) as within subjects’ factor and the W-moment as dependent variable.

To quantify whether mood induction sessions were effective in changing participants’ affective state, scores to each subscale (i.e., anger, fear, happiness, sadness) after each mood induction were compared to the respective baseline with paired comparisons. In addition, we also examined whether the different mood induction procedures led to different affective states by conducting four separate repeated measure ANCOVAs with each adjective scale as dependent variable and with the type of mood induction as within-subjects factor. For each analysis the baseline score to the relevant adjective scale was entered as covariate to control for possible differences in the baseline affective state.

For each analysis, Greenhouse–Geisser correction was used for sphericity, and the Bonferroni–Holm’s sequential correction for multiple comparisons was applied to the *post hoc* tests (Holm, 1979).

## Results

### Mood Induction Check

Paired comparisons between the baseline score and the score after each mood induction for each subscale are reported in **Table 1**. As compared to the baseline score, the induction of positive affect improved feelings of happiness, and at the same time reduced negative emotions such as fear and sadness. Conversely, the procedure employed to induce negative affect effectively increased negative emotions such as anger and sadness, and simultaneously decreased positive emotions such as happiness. Although the material used in the neutral condition was not meant to induce positive or positive emotions, we observed a significant attenuation of happiness in this condition, indicating that the stimulus material used in the neutral condition may not be emotionally neutral and may be perceived by participants as unpleasant. Conversely, no significant effects of the neutral mood induction were observed on the subscales measuring negative emotions.

**Table 1:** Mean scores and *SDs* to the four subscales measuring anger, fear, happiness, and sadness, at the beginning of the experiment – i.e., baseline – as well as after each mood induction – i.e., positive, neutral, and negative.

	Baseline	Mood induction					
		Positive		Neutral		Negative	
	Mean (*SD*)	Mean (*SD*)		Mean (*SD*)		Mean (*SD*)	
Anger	1.29 (0.56)	1.03 (0.11)	*t*(28) = 2.64 *p* = 0.013	1.18 (0.33)	*t*(28) = 1.11 *p* = 0.28	**2.67 (1.04)**	***t*(28) = -6.05 *p* < 0.0001 Cohen’s *d* = –1.16**
Fear	1.72 (0.58)	**1.22 (0.3)**	***t*(28) = 4.82 *p* < 0.0001 Cohen’s *d* = 0.97**	1.62 (0.87)	0.56, 0.58	2.05 (0.92)	–1.78, 0.086
Happiness	3.31 (0.77)	**4 (0.72)**	***t*(28) = -4.46 *p* < 0.0001 Cohen’s *d* = –0.83**	**2.77 (0.91)**	***t*(28) = 3.47 *p* = 0.002 Cohen’s *d* = 0.65**	**2.28 (0.72)**	***t*(28) = 6.63 *p* < 0.0001 Cohen’s *d* = 1.24**
Sadness	1.49 (0.68)	**1.16 (0.31)**	***t*(28) = 3.65 *p* = 0.001 Cohen’s *d* = 0.94**	1.33 (0.56)	*t*(28) = 1.31 *p* = 0.2	**2 (0.89)**	***t*(28) = –4.24 *p* < 0.0001 Cohen’s *d* = –0.83**

*The t and p values refer to paired comparisons between the corresponding condition and the baseline level for the same subscale. For comparisons that were significant after the Bonferroni–Holm’s sequential correction (in bold), Cohen’s d is also reported.*

**FIGURE 1:**
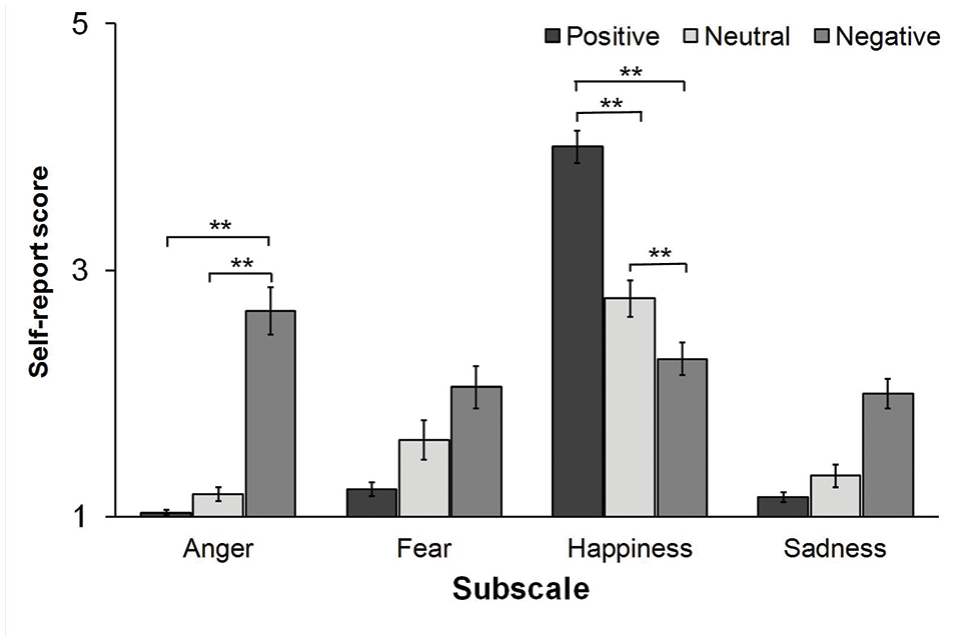
**Self-report scores to the adjective scales measuring anger, fear, happiness, and sadness after the positive, the neutral, and the negative mood inductions.** (^∗∗^*p* < 0.01).

While these observations indicate that the mood induction procedure effectively changed participants’ affective state, we also tested whether the reported affective state changed across conditions. The analyses revealed a main effect of the type of mood induction for the anger [*F*(2,54) = 14.51, *p* < 0.001, $\eta_{p}^{2}$ = 0.35] and the happiness [*F*(2,54) = 5.79, *p* = 0.009, $\eta_{p}^{2}$ = 0.18] subscales (see **Figure 1**). Planned comparisons indicated that participants scored higher on the anger subscale in the Negative condition (2.67 ± 1.03) as compared to both the Neutral (1.18 ± 0.33, *p* < 0.001, Cohen’s *d* = 1.51) and the Positive condition (1.03 ± 0.11, *p* < 0.001, Cohen’s *d* = 2.15). As for the happiness subscale, participants scored higher in the Positive condition (4 ± 0.72) as compared to the Neutral (2.76 ± 0.91, *p* < 0.001, Cohen’s *d* = 1.81) and the Negative condition (2.28 ± 0.72, *p* < 0.001, Cohen’s *d* = 1.85). Scores to the happiness subscale were also higher in the Neutral than in the Negative condition (*p* = 0.002, Cohen’s *d* = 0.72). No main effect of condition was found on self-report measures of fear and sadness (all *p*_s_ > 0.1).

Taken together, these observations indicate that both the Positive and the Negative mood inductions were effective in changing participants’ affective states. These changes were qualified as increased positive emotions (i.e., happiness) and reduced negative emotions (i.e., fear, sadness) after the positive mood induction. Conversely, the negative mood induction increased negative affect (i.e., anger, sadness) while simultaneously decreasing positive affect.

### Awareness of Intention

**FIGURE 2:**
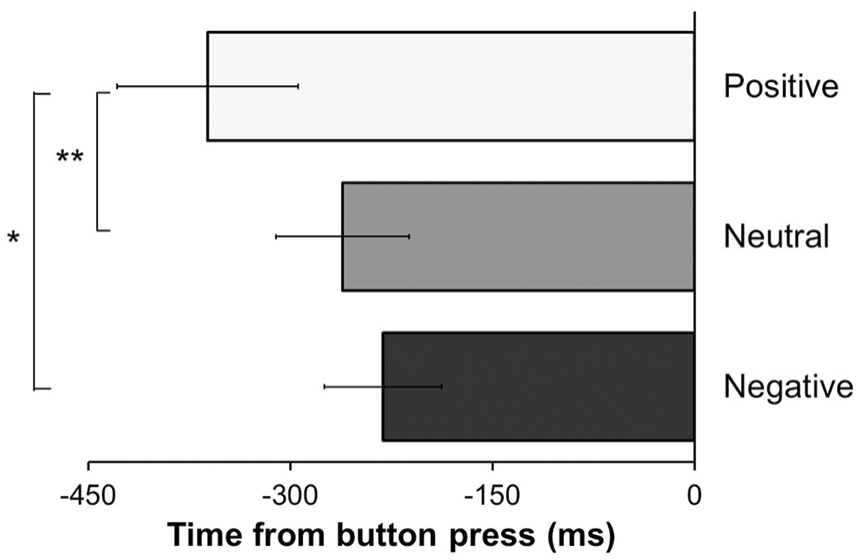
**Reported time (in milliseconds) of intention in relation to the button press – i.e., time 0.** Error bars indicate the SEM. (^∗^*p* < 0.05; ^∗∗^*p* < 0.01).

The analysis revealed a significant effect of the type of mood induction [*F*(1.51,42.14) = 3.63, *p* = 0.047, $\eta_{p}^{2}$ = 0.115], indicating that the W-moment varied across the experiment as function of the experimental condition. This effect was qualified by a significant linear trend, as revealed by significant linear contrasts analysis [*F*(1,28) = 4.66, *p* = 0.04, $\eta_{p}^{2}$ = 0.143], indicating a linear decrease of the W-moment across the three experimental conditions (see **Figure 2**). Planned comparisons revealed that W-moment were reported significantly earlier in time in the positive condition (361.42 ms ± 361.2) as compared to the neutral condition [261.24 ms ± 266.88; *t*(28) = 2.94, one-tailed *p* = 0.003; Cohen’s *d* = 0.32] as well as to the negative condition [231.2 ms ± 234.95; *t*(28) = 2.16, one-tailed *p* = 0.02; Cohen’s *d* = 0.43]. Conversely, W-moment did not differ between the negative and the neutral condition (*p* = 0.58).

We also wanted to test whether the induced affective states influenced the response time (RT) – i.e., the time interval between the onset of the trial and the participant’s button press – a repeated-measure ANOVA was conducted with condition as within-subjects variable and the individual averaged RT as dependent variable. The analysis revealed no effects of condition (*p* = 0.36).

## Discussion

The present study examined whether positive and negative affective states can impact on participants’ attention toward inner states of voluntary movement initiation. The mood induction procedure employed here effectively modulated participants’ affective state as measured by self-report adjective scales (see **Table 1**; **Figure 1**). Overall, participants reported more positive affect after being exposed to pleasant material and reported more negative affect after being exposed to unpleasant material. After each mood induction procedure participants were to perform the Libet task (Libet et al., 1983). In this task participants are asked to press a button at the time of their own choice and to report when they formed the intention to press the button. In line with our prediction, the intention to act was reported earlier in time – as indicated by the W-moment – after participants were primed with pleasant material as compared to when they were primed with unpleasant or neutral material (see **Figure 2**).

These results corroborate previous evidence that mood induction procedures combining guided imagery and music is an effective method to temporarily influence people’s affective states (Mayer et al., 1995; Vastfjall, 2002; Robinson et al., 2012). Exposure to pleasant auditory and written material effectively increased positive emotions, such as happiness, and decreased negative emotions such as sadness and fear, as compared to the pre-experiment affective state. Conversely, after being primed with unpleasant music and scenarios, participants reported more negative emotions such as anger and sadness and less happiness, as compared to a baseline level (see **Table 1**). Crucially, the patterns of reported emotions were considerably different after each mood induction session (see **Figure 1**). More precisely, the different types of mood induction led to differences in specific emotions, such as happiness and anger, while other emotions, such as sadness and fear, were comparable across conditions. This observation is in line with previous studies using the same pleasant and unpleasant stimulus material to specifically induce happiness and anger, respectively (Mayer et al., 1995). Since happiness and anger are similar in arousal but opposite to each other in valence (Russell, 1980; Posner et al., 2005), one can reasonably argue that the effects of the mood manipulation on intention awareness is driven by an increase of pleasant emotions in the Positive condition as compared to the Negative and the Neutral condition. However, it should be noted that anger and happiness also differ in motivational dimensions, with anger-related emotions being higher in approach motivation (Posner et al., 2005; Gable and Harmon-Jones, 2008) as compared to happiness. It is therefore possible that the changes in intention awareness reported here may be influenced also by motivational factors related to the approach-avoidance dimension, which was not controlled for in the current study.

These results are in line with our prediction that affective states can modulate intention awareness. Inducing positive mood leads to earlier W-judgments, which may reflect a broadening of the selective attention to inner states. Conversely, our prediction that negative affect would narrow intention awareness was not supported by the data. The reported time of intentions when participants were primed with anger-inducing material did not differ significantly from the neutral condition, that is, when participants were exposed to scenarios and music that was supposed to evoke neither positive nor negative affective states. However, it should be noted that participants reported a reduction in positive affect after the neutral induction (**Table 1**), indicating that the neutral condition elicited some emotional changes. Other control conditions (e.g., a different set of scenarios, different music, or neither scenarios nor music at all) might have garnered different results. Further research should therefore employ control conditions that allow for a clear distinction between neutral/baseline and negative mood.

These findings provide first evidence that affective states can influence intention awareness. One plausible interpretation of these findings is that positive affect, as compared to negative affect, enhances awareness of inner states related to action preparation and initiation, as measured by the W-judgment. Previous empirical studies showed that W-judgments can be modulated by a variety of factors, including neurological conditions, such as Parkinson’s disease (Tabu et al., 2015), tic disorder (Ganos et al., 2014), and lesions in the parietal cortex (Sirigu et al., 2004), stimulation of supplementary motor areas (Lau et al., 2007), and manipulations of the experimental stimuli, such as delayed sensory feedbacks (Banks and Isham, 2009; Rigoni et al., 2010). Here we show that W-judgments in healthy participants were modulated by a mood induction procedure designed to temporarily change participants’ affective state, suggesting that intention awareness is sensitive to people’s current emotional or mood state. In a way, this finding can be seen as an extension of previous findings (Aarts et al., 2012; Yoshie and Haggard, 2013) where it was shown that another component of intentional control, namely the sense of agency, was influenced in a similar way by positive affect. States of positive affects seem to boost the feeling of intentional control, as indicated by increased sense of agency (Aarts et al., 2012; Yoshie and Haggard, 2013) and by enhanced intention awarenss, as observed here and in previous studies (Jo et al., 2015).

The interpretation of the observed effects in terms of increased awareness critically relies on the assumption that the W-judgment is a valid marker of awareness of inner motor states. However, this assumption is still greatly debated (Gomes, 1998; Pockett, 2002; Banks and Isham, 2009). For instance, it is not possible to exclude that the mood induction affected participants’ implicit or explicit strategies to judge the time of their conscious decisions, rather than intention awareness itself. One alternative interpretation is that the induction of a positive mood increased visual awareness, which in turn may have impacted on the *post hoc* estimation of the time of the conscious intention when participants were to report the position of the dot on the clock. Given the subjective nature of the Libet task and of the W-judgment, it is not possible to exclude this alternative interpretation with the current data.

Our observation that positive affect leads to earlier awareness of intention is in line with a series of empirical studies showing that positive affect improves action control by boosting dopamine level in the brain (Ashby et al., 1999; Braver and Cohen, 2000; Cohen et al., 2002; Dreisbach, 2006). More precisely, it has been suggested that positive affect modulates cognitive control and cognitive flexibility by increasing the neurotransmitter dopamine in specific brain regions, namely in the striatum and the prefrontal cortex (Ashby et al., 1999). More recently, Cools et al. (2001, 2007) and Cools (2008) provided a refined version of this account by showing that the neural structure in which dopamine levels are sensitive to positive affect are indeed involved in the modulation of cognitive control. They reported evidence that increased dopamine in the striatum increased cognitive flexibility, while increased dopamine in the prefrontal cortex leads less distraction by irrelevant information. Interestingly, a recent study provides indirect evidence that the levels of dopamine do not only influence cognitive control, but also affect intention awareness (Tabu et al., 2015). By employing a modified version of the Libet task, the authors compared the reported time of the conscious intention in Parkinson’s patients without medication – therefore with dopamine depletion – with healthy participants, and found delayed intention awareness in those patients. While the Libet task employed here does not directly measure cognitive control, empirical evidence suggests that enhanced intention awareness is indeed associated with higher cognitive control. For instance, Ganos et al. (2014) found that tic patients with a higher ability to voluntarily control and suppress their tics also reported significantly earlier intention awareness, indicating that early detection of one’s motor states may indeed contribute to voluntary action control. On the one hand, the finding that positive affect leads to earlier intention awareness would therefore be in line with a neurophysiological account predicting increased dopamine level in relevant areas of the prefrontal cortex that are known to be crucial for volitional action control, such as the supplementary motor areas (Jenkins et al., 1992; Mattay et al., 2002; Lau et al., 2004). On the other hand, this observation may also have implications for conscious control of behavior (Libet, 1999). According to Libet (1999), although voluntary actions are initiated unconsciously – as indicated by the early onset of motor-related brain potentials – the individual can still modify or inhibit the action once movement preparation reaches awareness (Libet, 1999, 2006). This capacity to *veto* the action, referred to as *free won’t* (Obhi and Haggard, 2004), would be the crucial function of our “conscious will” (Libet, 1999). Our finding would therefore imply that positive affect may provide additional time to the conscious will to modify the action, by widening the temporal interval between the moment at which we become aware of movement initiation and the actual movement. However, as described previously, the limitations of the Libet method for the investigation of the conscious intention call for a cautious interpretation of our data in terms of enhanced awareness and conscious control.

While we provide evidence that positive affect influences awareness of motor intentions, our hypothesis that negative affect would narrow attentional breadth toward inner intentional states was not confirmed by our data. There are, in our view, two plausible explanations that may account for this lack of effect of negative mood on intention awareness. One possibility is that the mood induction procedure was less effective in triggering negative affect as it was in triggering positive affect. Although self-report data indicate that the mood induction procedure indeed increased anger-related affect, it is possible that mood induction manipulation was not strong enough to affect neurocognitive processes underlying intention awareness. An alternative interpretation is that brain processes that underlie intention awareness are not sensitive to negative mood manipulations. While positive affect is assumed to influence cognition – and executive functions more specifically – mainly by increasing dopamine levels in the brain (Ashby et al., 1999, 2002; Fredrickson, 2004; Mitchell and Phillips, 2007), the impact of negative mood on the same cognitive functions is much less defined. More precisely, the effect of negative affect is likely to be mediated by different neurotransmitters as compared to those involved in positive affect, such as serotonin levels, that have only little impact on executive functions (for a review see Mitchell and Phillips, 2007). It is therefore possible that intention awareness, as measured by the Libet task, is relatively unrelated to changes in serotonin level due to negative mood induction.

## Conclusion

This study was the first to show that intentional awareness is influenced by the affective state participants are in. Our observations nicely fit with the idea that positive emotional states broadens selective attention and adds to it that it can redirect participants’ attention toward inner states of voluntary movement initiation. However, given the limitations of the Libet task, the interpretation of our data in terms of increased intentional awareness should be taken with caution. Further research should therefore provide converging evidence that positive affect, as compared to neutral or negative emotions, enhances intentional action control by employing different tasks and paradigms. In addition, future studies should investigate how intention awareness is influenced by different types of both positive and negative emotions while controlling for valence, arousal, and other motivational dimensions such as approach and withdrawal. In our opinion, this line of research would crucially elucidate how conscious control is linked to specific emotional states or moods, and would therefore be of relevance for several domains, such as health and clinical psychology as well as for forensic settings.

## Conflict of Interest Statement

The authors declare that the research was conducted in the absence of any commercial or financial relationships that could be construed as a potential conflict of interest.
